# Germline *TP53* mutation spectrum in Sudanese premenopausal breast cancer patients: correlations with reproductive factors

**DOI:** 10.1007/s10549-019-05168-1

**Published:** 2019-02-22

**Authors:** Gitana Maria Aceto, Khalid Dafaallah Awadelkarim, Marta Di Nicola, Carmelo Moscatello, Mattia Russel Pantalone, Fabio Verginelli, Nasr Eldin Elwali, Renato Mariani-Costantini

**Affiliations:** 1 Department of Medicine, Dentistry and Biotechnology, “G. d’Annunzio” University, Via dei Vestini 31, 66100 Chieti, Italy; 2 Department of Molecular Biology, National Cancer Institute (NCI-UG), University of Gezira, P. O. Box 20, Wad Medani, Sudan; 3 Division of Microbial Pathogenesis, Department of Medicine (Solna), BioClinicum, Karolinska Institutet, 17164 Stockholm, Sweden; 4 Department of Pharmacy, “G. d’Annunzio” University, Via dei Vestini 31, 66100 Chieti, Italy; 5 Unit of General Pathology, Aging and Translational Medicine Research Center (CeSI-MeT), “G. d’Annunzio” University, Via Luigi Polacchi 11, 66100 Chieti, Italy; 6 Department of Biochemistry, Faculty of Medicine, Imam Muhammad ibn Saud Islamic University, 7544 - Othman Bin Affan Rd. Al-Nada, Riyadh, 13317-4233 Saudi Arabia

**Keywords:** Breast cancer, Sub-Saharan Africa, *TP53*, Germline mutation, Reproductive factors

## Abstract

**Purpose:**

The role of non-genetic factors as modifiers of *TP53*-related hereditary breast cancer (BC) risk is debated. In this regard, little is known about the impact of germline *TP53* mutations on BC in sub-Saharan Africa, where the disease often presents in non-contraceptive multiparous premenopausal women with extended history of breastfeeding. Herein, we report the germline *TP53* mutations found in a series of 92 Sudanese premenopausal BC patients characterized for reproductive history.

**Methods:**

The entire *TP53* coding sequence, including intron–exon boundaries and UTRs, was analyzed via DHPLC and direct sequencing, and the association of *TP53* genotypes with BC risk and with individual lifetime exposures to reproductive factors was investigated with statistical tools.

**Results:**

The germline *TP53* mutation spectrum comprised 20 variants, 15 in the non-coding and 5 in the coding region. The latter included a deleterious missense mutation, c.817C>T (p.Arg273Cys), in a unique patient, and the common and functionally relevant coding polymorphism at amino acid 72 [Pro72Arg (rs1042522)]. The non-coding mutations included c.919+1G>A, a known deleterious splice site mutation, also in a unique patient. Notably, the 2 carriers of deleterious *TP53* mutations clustered in the subset of cases with stronger reproductive history relative to childbearing age. When analyzed in comparison to population controls, the codon 72 polymorphism did not reveal associations with BC.

**Conclusions:**

Our study suggests that the codon 72 Arg>Pro polymorphism is not implicated in premenopausal BC susceptibility, whereas multiparity and breastfeeding might be BC risk factors for carriers of deleterious *TP53* mutations.

**Electronic supplementary material:**

The online version of this article (10.1007/s10549-019-05168-1) contains supplementary material, which is available to authorized users.

## Introduction

Breast cancer (BC) is the most prevalent type of tumor in women who carry a deleterious germline *TP53* mutation in the setting of the Li–Fraumeni syndrome (LFS, OMIM #151623) [1]. However, putatively deleterious germline *TP53* mutations (OMIM #191170) are also associated with less prominent cancer phenotypes and, in genome-wide studies, seem to be more frequent than the estimated prevalence of the LFS [2]. In fact, *TP53* mutations are found in ~ 2–3% of premenopausal BC patients unselected for the LFS criteria [3] and in < 1% to ~ 7% of the early onset BC cases (age < 30 years) in population-based series [4]. This suggests that non-genetic factors modify *TP53*-related inherited BC risk. In this regard, it is well known that reproductive history and exogenous exposure to female hormones are the most consistent modifiers of BC risk after genetic predisposition and age [5].

In Africa, BC often presents in multiparous women in the middle of their childbearing years, who are mostly unexposed to hormonal contraceptives and generally report long lifetime duration of breastfeeding [6–8]. Hence, the characterization of *TP53* carriers in sub-Saharan African BC series might shed some light on the role of reproductive history as a modifier of *TP53*-related hereditary BC risk.

The majority of the genetic studies about BC in sub-Saharan Africa is focused on the analysis of the mutational spectra of the *BRCA1*/*2* genes [9]. With regard to the *TP53* gene, two cases of female BC in native sub-Saharan African mutation carriers were recently reported within a familial LFS context [10], but, to our knowledge, comprehensive data on germline *TP53* variation in sub-Saharan African BC case series are still lacking.

Here, we describe the spectrum of germline *TP53* mutations found in a mono-institutional Sudanese premenopausal BC series comprising 92 cases, that were consecutively recruited between 1999 and 2004, when the Sudanese fertility rate ranged from 5.54 to 5.20 [11–13].

## Materials and methods

### Patients and controls

The study was approved by the Institutional Ethical Committees of the National Cancer Institute, University of Gezira (NCI-UG) and “G. d’Annunzio” University. The 92 pathologically confirmed BC patients, all diagnosed within 45 years of age (mean age: 36.3 ± 5.9 years; range: 23–45 years) had been selected on a consecutive basis among the BC patients treated at NCI-UG from May 1999 through December 2004, as we previously described [11, 12]. Medical records were reviewed with regard to individual, pathological, clinical, and reproductive data (including self-reported information on age at menarche). Hormone receptor data were not available, due to poor fixation and storage of tissue blocks, a problem that we already highlighted [6]. A group of 180 control subjects (111 males and 69 females) was recruited from the Wad Medani city region (mean age 36.1 ± 10.4 years, range 20–70 years; male/female ratio 111/69; mean age of females 38.4 ± 10.1 years, range 20–65 years; mean age of males 34.7 ± 10.4 years; range 20–70 years). Due to the limited amounts of DNA, variable numbers of these controls were used to verify variant frequencies. Acid citrate–dextrose anticoagulated blood (10 ml) was collected from each patient and control and preserved at − 20 °C in aliquots. All patients and controls gave informed consent to the study, according to guidelines set by the NCI-UG.

#### TP53 mutation analysis

Genomic DNA was isolated from peripheral blood using the QIAamp DNA Blood Mini Kit (QIAGEN, Chatsworth, CA). The entire *TP53* coding sequence (NM_000546), including intron–exon boundaries and UTRs, was analyzed via DHPLC using a Wave^®^Nucleic Acid Fragment Analysis System (Transgenomic Inc., San Jose, CA). To detect homozygous variants, the PCR-amplified samples were tested also after mixing with an aliquot of known wild-type DNA (2µl:5 µl). Samples with altered DHPLC profiles were directly sequenced using an ABI PRISMTM3100 genetic analyzer (Applied Biosystems, Foster City, CA). Primers for *TP53* exons 1–11 were reported by Verselis et al. [14], those used for UTRs are listed in the online resources (Table ESM_1). Variants were always confirmed in replicate assays. Nomenclature follows the guidelines of the Human Genome Variation Society (HGVS, http://www.HGVS.org/varnomen), clinical significance and geographic distribution were assessed using ClinVar (https://www.ncbi.nlm.nih.gov/clinvar/), the International Agency for Research on Cancer (IARC) *TP53* database [15], MutPred2 [http://mutpred.mutdb.org], Ensemble (1000 Genomes Project, phase 3) [https://www.ensembl.org/] and COSMIC [https://cancer.sanger.ac.uk/cosmic]. Variants with minor allele frequency (MAF) ≥ 1% were considered neutral [16], those with MAF < 1% and deleterious mutations were searched in healthy controls.

#### Statistical analyses

A logistic regression model was applied to test the association of *TP53* genotypes and haplotypes with BC risk. Results were expressed as odds ratio (OR) and relative 95% confidence interval (95% CI). For each polymorphism with minor allele frequency (MAF) ≥ 5%, the Hardy–Weinberg equilibrium (HW) was tested using the Hardy–Weinberg calculator (http://ihg.gsf.de/cgi-bin/hw/hwa1.pl). The threshold of statistical significance was set at *p* = *0.05*. Data analysis was performed using SPSS Advanced Statistics TM 13 (2004, Chicago, IL, USA).

To approximate individual lifetime exposures to reproductive factors, we developed reproductive factor scores for each patient meeting verifiable data requirements (age at menarche, number of pregnancies, and reported breastfeeding of infants). Given the difficulty of obtaining precise information on the individual duration of the breastfeeding periods, we considered a period of 24 months for each child, based on the frequency of extended breast feeding at 2 years of age in rural Sudan for the period during which the BC cases were recruited [17]. The reproductive factor scores were generated by subtracting the total period in months estimated to be occupied by pregnancies and breastfeeding to the cumulative number of fertile months calculated from menarche to BC diagnosis. Relative to the median, the factor score values ranged from negative (−) to positive (+), thus identifying 2 subsets of patients, respectively, defined by stronger (−) versus weaker (+) reproductive history relative to childbearing age. Fisher’s exact test was used to analyze the significance of the differences in the frequencies of deleterious mutations and codon 72 genotypes (homozygous and heterozygous) between the 2 subsets of cases defined by reproductive factor score values.

## Results and discussion

We characterized for germline *TP53* mutations 92 female BC patients selected based on diagnosis within 45 years of age. Overall, we found 20 germline *TP53* variants (Table 1), of which 5 in the coding region (2 missense, including a deleterious mutation plus a common coding polymorphism and 3 synonymous) and 15 in the non-coding region (6 intronic, including 5 polymorphisms plus a known deleterious splice site mutation, and 9 in the UTRs, of which 5 were known polymorphisms and 4 novel). The findings were consistent with the expected higher frequency of non-coding sequence variation [15, 16, 18].

**Table 1:** Germline *TP53* variants identified in 92 premenopausal Sudanese breast cancer patients diagnosed within age 45

*TP53* mutation	Effect	Amplicon	SNP ID	Frequency		Clinical significance
				Cases	Controls	
Missense						
c.215C>G	p.Pro72Arg	4	rs1042522	48/184 (26%)	60/232(26%)	Benign/uncertain
c.817C>T	**p.Arg273Cys**	**8**	**rs121913343**	**1**/**184 (0.5%)**	**0**/**114**	**Pathogenic**
Synonymous						
c.108G>A	p.Pro36Pro	4	rs1800370	2/184 (1.1%)	4/232 (1.7%)	Benign
c.555C>T	p.Ser185Ser	5	rs367560109	1/184 (0.5%)	0/114	Benign
c.639A>G	p.Arg213Arg	6	rs1800372	3/184 (1.6%)	2/114 (1.8%)	Benign
Intronic						
c.96+56ins16	STR	3	rs17878362	30/166 (18%)	26/128 (20%)	NA
c.782+17C>T	–	7	rs17880172	3/184 (1.6%)	0/114	Benign
c.919+1G>A	**Splice**	**8**	**NA**	**1**/**184 (0.5%)**	**0**/**114**	**Pathogenic**
c.993+12T>C	–	9	rs1800899	3/184 (1.6%)	0/114	Benign
c.1101−49C>T	–	11	rs17881850	5/184 (2.7%)	NE	Benign
c.1101−73G>C	–	11	rs17883043	1/184 (0.5%)	0/114	Benign
UTR						
c.1−10788G>C	–	1	Novel	1/184 (0.5%)	0/114	Novel
c.75−32C>A	–	2	Novel	1/184 (0.5%)	0/114	Novel
c.74+38G>C	–	2	rs1642785	38/170 (22.4%)	29/100 (29%)	Benign
c.75−40G>T	–	3	Novel	1/184 (0.5%)	0/114	Novel
c.672+62A>G	–	6	rs1625895	32/184 (17.2%)	47/208 (22.6%)	Benign
c.*+267G>A	–	11.2	Novel	1/184 (0.5%)	0/114	Novel
c.*+569−70delGT	–	11.3	rs17886358	5/184 (2.7%)	NE	NA
c.*+826G>A	–	11.5	rs17884306	2/184 (1.1%)	NE	Likely benign
c.*+1070C>T	–	11.6	rs114831472	2/184 (1.1%)	NE	Likely benign

Frequencies were calculated by n/2N (*n* number of minor alleles, *N* number of screened chromosomes), clinical significance is interpreted according to ClinVar (https://www.ncbi.nlm.nih.gov/clinvar/) and to COSMIC (https://cancer.sanger.ac.uk/cosmic). *STR* short tandem repeat

The two deleterious mutations were c.817C>T (p.Arg273Cys), detected in a patient diagnosed with BC at age 32 (case S6), and (c.919+1G>A), detected in a patient diagnosed at age 30 (case S13). No reliable family history data were retrievable for these cases, as is usually the case in rural sub-Saharan Africa [7]. The p.Arg273Cys mutation, located in the DNA-binding domain, is reported with both somatic (706-fold) and germline (35-fold) status in the TCAG [http://dgv.tcag.ca] and IARC [http://www-p53.iarc.fr/; R19] *TP53* databases. The c.919+1G>A mutation, that targets the splice donor site of intron 8, is also reported in the IARC database as germline (sixfold) and somatic (13-fold). Thus, the prevalence of identified deleterious *TP53* mutations was 2.17% (2/92), i.e., comparable to that found in Western premenopausal BC series [3]. Ours is likely an underestimate, because we did not investigate regulatory elements outside the UTRs. Besides, our screening techniques, highly sensitive for point mutations and small insertions/deletions (the most common *TP53* mutation types), do not detect genomic rearrangements.

The 4 novel UTR variants were found in individual patients and not in control chromosomes from the Wad Medani population (Table 1). It cannot be excluded that these variants could influence *TP53* mRNA stability and translational efficiency, but their evaluation requires functional assays, which are beyond the scope of the present report.

Apart from the above mentioned, the remaining 14 variants, found in several cases, correspond to known polymorphisms (Table 1). Still, polymorphisms may affect TP53 protein level or functions, especially when altering the coding sequence, and could thus influence BC risk. In particular, there is a debate on the common *TP53* polymorphism at amino acid 72 [Pro72Arg (rs1042522)], here referred to as P72 and R72, which was one of the 4 most frequent polymorphisms in our case series (Table 1) [19, 20]. The P72 variant, that has weaker transcriptional activity and lower apoptotic potential [21, 22], is linked to poor prognosis after adjuvant therapy, rather than to BC risk [23], while R72 is associated with increased mitochondrial function, metastatic capability [24], and higher affinity for the E6 oncoprotein of the human papillomaviruses [25]. In this regard, it was reported that Sudanese BC patients show an excess of homozygous R72 compared to controls [26]. However, in the present case series, the frequencies of P72 and R72 (RS1042522) did not significantly differ between cases and controls, which does not support an association of this polymorphism with premenopausal BC risk (Table 2).

**Table 2:** Genotype and allele frequencies of the *TP53* variants with minor allele frequencies ≥ 5% in breast cancer patients and controls

refSNP ID#	Variant	Genotype	Frequency (%)		OR (95% CI)	*p* value
			Cases	Controls		
rs1642785	c.74+38G>C	G/G^a^	43 (46.7)	17 (29.8)		
		G/C	43 (46.7)	31 (54.4)	0.55 (0.26–1.14)	0.105
		C/C	6 (6.5)	9 (15.8)	0.26 (0.08–0.85)	0.026
	Alleles	G^a^	129 (70.1)	65 (57.0)		
		C	55 (29.9)	49 (43.0)	0.56 (0.35–0.92)	0.022
H–W (*p* value)			0.269	0.408		
rs17878362	c.96+56ins16	–/–^a^	46 (50.0)	36 (37.1)		
		–/ins	34 (37.0)	40 (41.2)	0.66 (0.35–1.25)	0.206
		ins/ins	12 (13.0)	21 (21.6)	0.45 (0.19–1.02)	0.058
	Alleles	–^a^	126 (68.5)	112 (57.7)		
		ins	58 (31.5)	82 (42.3)	0.63 (0.41–0.96)	0.031
H–W (*p* value)			0.167	0.127		
rs1042522	c.215C>G	C/C^a^	23 (25.0)	35 (30.2)		
		C/G	47 (51.1)	60 (51.7)	1.19 (0.62–2.28)	0.596
		G/G	22 (23.9)	21 (18.1)	1.59 (0.72–3.53)	0.251
	Alleles	C^a^	93 (50.5)	130 (56.0)		
		G	91 (49.5)	102 (44.0)	1.25 (0.84–1.83)	0.265
H–W (*p* value)			0.834	0.592		
rs1625895	c.672+62A>G	G/G^a^	48 (52.2)	24 (38.1)		
		G/A	34 (37.0)	30 (47.6)	0.57 (0.28–1.13)	0.108
		A/A	10 (10.9)	9 (14.3)	0.55 (0.20–1.55)	0.261
		G^a^	130 (70.6)	78 (61.9)		
		A	54 (29.4)	48 (38.1)	0.67 (0.42–1.09)	0.108
H–W (*p* value)			0.296	0.939		

*p* ≤ 0.05 was considered statistically significant

*OR* odds ratio, *CI* confidence interval, *H–W* Hardy–Weinberg equilibrium; ^a^Reference

The other 3 polymorphisms with MAF ≥ 5% were c.74+38G>C (rs1642785), c.96+56 ins16 (rs17878362), reportedly linked to P72 and associated with cancer risk in non-Africans [27], and c.672+62A>G (rs1625895) (Tables 1, 2). The frequencies of these variants, verified in population controls (Table 2), were in agreement with the Hardy–Weinberg equilibrium for c.672+62A>G (Table 2) and, rather than showing association with BC, were unbalanced towards controls for both c.74+38G>C (*p* = 0.022) and c.96+56 ins16 (*p* = 0.031).

In the general female population, early parity is associated with decreased long-term risk of BC, however, regardless of age at delivery, the risk slightly and transiently increases in the first 3–5 years after childbirth [28]. At present, there is still a lack of data concerning the influence of reproductive history on BC risk in carriers of *TP53* mutations. Experimental evidence from a mouse model shows that parity protects p53-deficient mice from developing mammary preneoplasia [29].

**Fig. 1:**
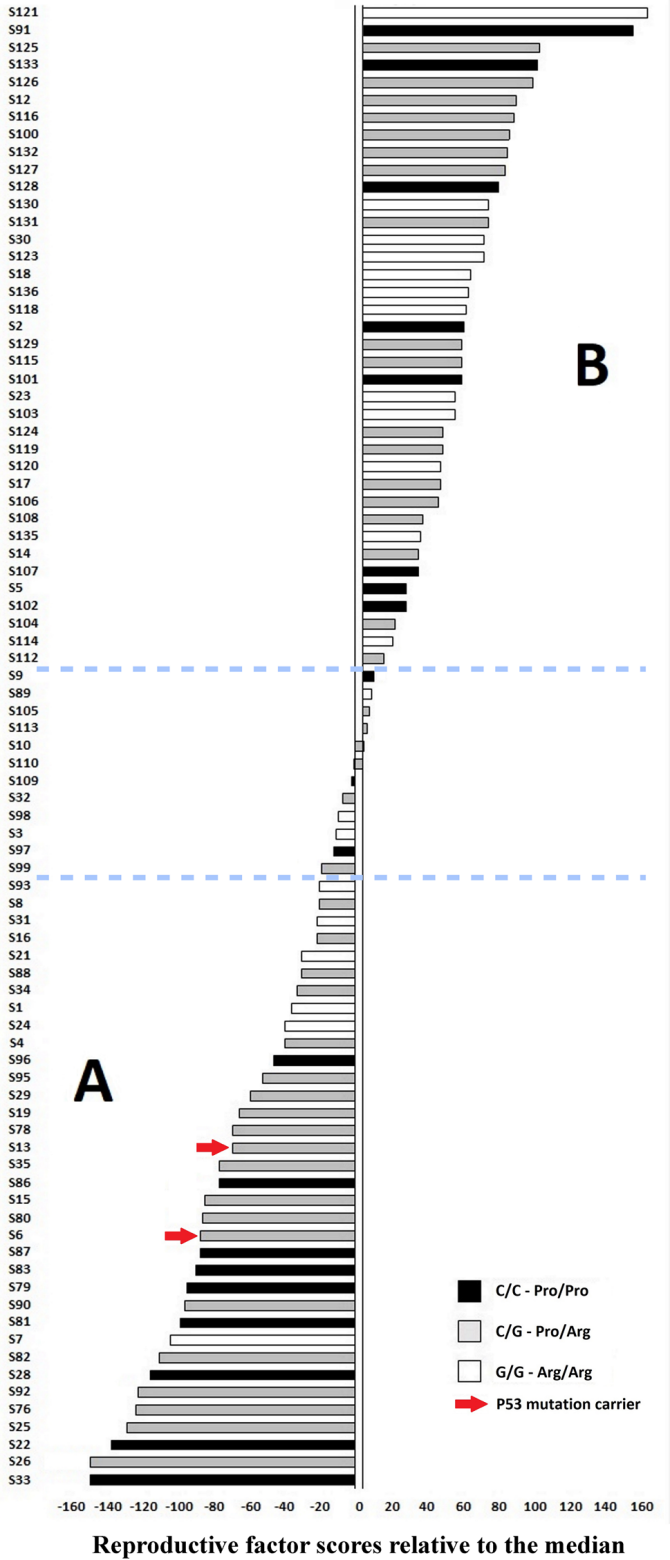
Reproductive factor scores expressed in terms of individual histograms for 85 breast cancer patients. Relative to the median, the factor scores differences ranged from − 148.49 to + 160.09 and identified 2 subsets of cases, designated **A** (35 cases, lower 95% CI of mean from − 148.49 to − 19.71) and **B** (38 cases, upper 95% CI of mean from 11.34 to 160.09), respectively, with stronger (**A**) and weaker (**B**) reproductive history. The 12 cases between the blue dashed lines fall outside the CIs. The two carriers of deleterious TP53 mutations (red arrows) are in cluster **A**. Codon 72 heterozygotes (gray histograms) are more numerous in subset **A**, but without statistical significance. Homozygous carriers of P72 (black histograms) and R72 (white histograms) split between the two subsets

In the currently studied BC series, the available information allowed to create reproductive factor scores for 85/92 cases. These scores were based on the estimated cumulative number of months not occupied by pregnancy and breastfeeding during the fertile period up to breast cancer diagnosis. Relative to the median value (267), the factor score differences ranged from − 148.49 to + 160.09 and identified 2 subsets of cases, designated A (35 cases, lower 95% CI of mean from − 148.49 to − 19.71) and B (38 cases, upper 95% CI of mean from 11.34 to 160.09), defined by stronger (subset A) versus weaker (subset B) reproductive history adjusted for childbearing age (Fig. 1). The two carriers of deleterious *TP53* mutations, S6, c.817C>T (p.Arg273Cys), age 32, and S13 (c.919+1G>A), age 30, respectively, reporting 6 and 4 live births before BC diagnosis, joined subset A (individual reproductive factor scores: − 86.77 and − 68.77, respectively). This suggests that, differently from the murine model [29], parity and breastfeeding could be harmful for *TP53* mutation carriers. The codon 72 polymorphism did not reveal relevant associations with reproductive history, except a non-significant excess of codon 72 heterozygotes in subset A (Fig. 1).

## Conclusions

In conclusion, this study shows that in our Sudanese context, the common and debated polymorphism at codon 72 is not associated with premenopausal BC, and suggests that parity and breastfeeding represent BC risk factors for *TP53* mutation carriers. Given the relatively small size of our case series, our findings should be interpreted with caution and confirmatory studies in Sudan and other sub-Saharan African countries are needed.

## Electronic supplementary material

Below is the link to the electronic supplementary material.

Supplementary material 1 (PDF 98 KB)
