# Supplementary material for: Germline TP53 mutation spectrum in Sudanese premenopausal breast cancer patients: correlations with reproductive factors
Source: Breast Cancer Res Treat. 2019 Feb 22;175(2):479–85. doi: 10.1007/s10549-019-05168-1 (PMC6533225; doi:10.1007/s10549-019-05168-1)
Supplement: Supplementary file 1 — Supplementary material 1 (PDF 98 KB) [file 10549_2019_5168_MOESM1_ESM.pdf]

Table S1. Primers for *TP53* UTR used for the discrimination of SNPs with minor allele frequency >5%.

Oligo	Amplicon	*Forward (5'→ 3')*	*Reverse (5'→ 3')*
P53-1	5'UTR	CAACTCCATTTCCTTTGCTTC	GTGACTCAGAGGACTCAT
P53-11-1	3'UTR	CTGACTCAGACTGACATTCT	CTAAGCTGGTATGTCCTACT
P53-11-2	3'UTR	CCTGCACAGGTGTTTTGTTGT	TTCATTAACCCTCACAATGCAC
P53-11-3	3'UTR	TGTGAAATGCTGGCATTTGCA	TCACCAAGAGGTTGTCAGACA
P53-11-4	3'UTR	GGTAGAGGGAGTTGTCAAGT	GCAACAAAGCGAGACCCAGT
P53-11-5	3'UTR	TGATCTGGATCCACCAAGAC	ATGCAAAAGTTGGCTGGCCAT
P53-11-6	3'UTR	TAGCTGGGACCACAGGTTCAT	TGCAGATGTGCTTGCAGAATGT
P53-11-7	3'UTR	TGCTGGGATTACAATTGTGAG	ACACTCATTGCAGACTAGGT

SNP	Amplicon	*Forward (5'→ 3')*	*Reverse (5'→ 3')*
rs1642785	2-3	TGGAAGTGTCTCATGCTGGAT	GAGCAGTCAGAGGACCAGGT
rs17878362	3	CTGAAAACAACGTTCTGGTA	AAGGGGGACTGTAGATGGGTG
rs1042522	4	TTGATGCTCCCCGGACGA	TCATGGAAGCCAGCCCCTCA
rs1625895	6	GACAGGGCTGGTTGCCCA	ACTACAGGTGCCCAGCACCA

Article title: Germline TP53 mutation spectrum in Sudanese premenopausal breast cancer patients: correlations with reproductive factors

Journal name: Breast Cancer Research and Treatment

Author names: Gitana Maria Aceto, Khalid Dafaallah Awadelkarim, Marta Di Nicola, Carmelo Moscatello, Mattia Russel Pantalone, Fabio Verginelli, Nasr Eldin Elwali, Renato Mariani-Costantini

Affiliation and e-mail address of the corresponding author:

Renato Mariani-Costantini

Department of Medicine, Dentistry and Biotechnology, "G. d'Annunzio" University, Via dei Vestini 1, 66100 Chieti, Italy;

Unit of General Pathology, Aging and Translational Medicine Research Center (CeSI-MeT), "G. d'Annunzio" University, Via Luigi Polacchi 11, 66100 Chieti, Italy; rmc@unich.it

rmc@unich.it
